# Dapagliflozin-Induced Acute Pancreatitis: A Case Report and Review of Literature

**DOI:** 10.1155/2020/6724504

**Published:** 2020-02-14

**Authors:** Sunam M. Sujanani, Mohanad M. Elfishawi, Paria Zarghamravanbaksh, Francisco J. Cuevas Castillo, David M. Reich

**Affiliations:** Department of Medicine, Icahn School of Medicine at Mount Sinai, NYC Health and Hospitals/Queens, Jamaica, NY 11432, USA

## Abstract

Sodium-glucose cotransporter 2 (SGLT2) inhibitors are increasingly used as add-on therapy in patients with poorly controlled type 2 diabetes mellitus (T2DM). Although pancreatitis is not a known side effect of SGLT-2 inhibitors, there have been case reports of SGLT-2 inhibitor use being associated with pancreatitis. *Case Presentation*. A 51-year-old male with a history of type 2 diabetes, dyslipidemia, and status-post cholecystectomy presented to the emergency room with a four-day history of periumbilical pain radiating to the back. He denied any history of recent alcohol intake or prior episodes of pancreatitis. On physical examination, his abdomen was diffusely tender to palpation without guarding or rebound. Initial labs were notable for a leukocyte count of 9.3 × 10^9^/L, creatinine level of 0.72 mg/dL, calcium level of 9.5 mg/dL, lipase level of 262 U/L, and triglyceride level of 203 mg/dL. His last HbA1c was 8.5%. CT scan of his abdomen and pelvis showed findings consistent with acute pancreatitis with no biliary ductal dilatation. Careful review of his medications revealed the patient was recently started on dapagliflozin five days prior to admission in addition to his longstanding regimen of insulin detemir, sitagliptin, metformin, and rosuvastatin. His symptoms resolved after discontinuation of sitagliptin and dapagliflozin. A year later, due to increasing HbA1c levels, a decision was made to rechallenge the patient with dapagliflozin, after which he developed another episode of acute pancreatitis. His symptoms resolved upon cessation of dapagliflozin. *Conclusion*. This case highlights the possible association of SGLT-2 inhibitors and pancreatitis. Patients should be informed about the symptoms of acute pancreatitis and advised to discontinue SGLT-2 inhibitors in case such symptoms occur.

## 1. Introduction

Type 2 diabetes mellitus (T2DM) is the most common cause of end-stage renal failure requiring hemodialysis and is the seventh leading cause of mortality in the United States [1]. Several treatment options are used in the management of T2DM including insulin, metformin, glucagon like peptide-1 (GLP-1) receptor agonists, dipeptidyl peptidase-4 (DPP-4) inhibitors, and sodium-glucose cotransporter-2 (SGLT-2) inhibitors [2].

SGLT-2 inhibitors are a novel class of diabetes medications that work by decreasing glucose reabsorption in the kidney, thereby increasing renal glucose elimination and reducing blood glucose levels [3].

Empagliflozin, dapagliflozin, canagliflozin, and ertugliflozin are members of the SGLT-2 inhibitor class that have been approved by the Food and Drug Administration (FDA) [4]. SGLT-2 inhibitors are increasingly being used as add-on therapy in patients with inadequately controlled T2DM [2, 5]. In addition, empagliflozin and canagliflozin have also been shown to improve cardiovascular and renal outcomes in patients with T2DM [6–9]. Interestingly, dapagliflozin was shown to reduce the risk for congestive heart failure exacerbation [10, 11]. Adverse effects reported with this class of medications include genitourinary tract infections, urinary frequency, and rarely euglycemic diabetic ketoacidosis (DKA) [12, 13].

This report illustrates a case of acute pancreatitis precipitated by the addition of dapagliflozin to long-standing sitagliptin-metformin therapy and highlights the possible association between SGLT-2 inhibitors and pancreatitis.

## 2. Case Report

A 51-year-old Hispanic male with a past medical history significant for T2DM, dyslipidemia, and cholecystitis status-post cholecystectomy seven years ago presented to the emergency room with a four-day history of periumbilical pain radiating to the back along with nausea and decreased appetite. He denied any history of recent alcohol intake, autoimmune disorders, or prior episodes of acute pancreatitis. He is an active smoker (five cigarettes per day since thirty years).

On presentation, vital signs included a temperature of 36.4°C (97.5°F), pulse of 77 beats per minute, blood pressure of 155/96 mm Hg, respiratory rate of 18, and oxygen saturation of 100% on room air. Physical examination was remarkable for tenderness to light palpation diffusely in his abdomen with no guarding or rebound. Initial labs were notable for a leukocyte count of 9.3 × 10^9^/L, serum creatinine level of 0.72 mg/dL, serum calcium level of 9.5 mg/dL, serum lipase level of 262 U/L, and serum triglyceride level of 203 mg/dL. His last hemoglobin A1c one month prior to presentation was 8.5%. CT scan of his abdomen and pelvis showed status-post cholecystectomy with no biliary ductal dilatation, along with findings consistent with acute pancreatitis. Patient was placed nil per os (NPO), and outpatient oral medications were held. He was managed with IV fluids, antiemetics, and insulin.

Careful review of his medications revealed that the patient was started on dapagliflozin 10 mg daily five days prior to admission in addition to his long-standing regimen of insulin detemir 20 units twice daily, sitagliptin-metformin 50–1000 mg twice daily, and rosuvastatin 20 mg daily. The patient's symptoms improved within two days, and his diet was advanced. Upon discharge, insulin, metformin, and rosuvastatin were resumed. Dapagliflozin and sitagliptin were discontinued in light of the episode of pancreatitis. One month after discharge, glimepiride was added to his regimen by his endocrinologist.

Three-month, six-month, and nine-month follow-up visits revealed no further episodes of pancreatitis, but due to increasing hemoglobin A1c levels and lack of ample evidence of dapagliflozin precipitating pancreatitis, a decision was made to rechallenge the patient with dapagliflozin 10 mg in addition to continuing the concurrent regimen of insulin detemir, metformin, and glimepiride. The patient presented to the emergency room seven days after initiation of dapagliflozin with complaints of three-day history of nausea and epigastric pain radiating to the back. Laboratory findings were notable for a leukocyte count of 8.9 × 10^9^/L and serum lipase level of 138 U/L. CT scan of his abdomen and pelvis revealed findings suggestive of acute pancreatitis. Dapagliflozin was subsequently discontinued with resolution of symptoms. There has been no recurrence of pancreatitis since discontinuation of dapagliflozin.

## 3. Discussion

SGLT-2 inhibitors are being considered as possible causes of pancreatitis. Review of literature shows seven published case reports of acute pancreatitis attributed to SGLT-2 inhibitors. Four of them were associated with canagliflozin, two with empagliflozin, and one with dapagliflozin (Table 1).

Among these seven studies, the mean age of patients is 52.8 (range from 33–71), three were males, and four were females. The time interval between the initiation of SGLT-2 inhibitors and pancreatitis was 7 days with dapagliflozin, 10–84 days with canagliflozin, and 30–104 days with empagliflozin. In our case report, the time interval was around 5 days after initiation of dapagliflozin in the initial presentation and subsequent presentation of pancreatitis upon rechallenge with dapagliflozin.

Metformin was listed as a concomitant diabetes medication in all published case reports with canagliflozin and dapagliflozin and one with empagliflozin. One possible mechanism could involve dehydration and lactic acidosis, leading to pancreatitis [21].

In this report, the patient presented with a picture of acute pancreatitis in the setting of newly introduced dapagliflozin in the absence of a known cause of pancreatitis such as alcohol or gallstones. Furthermore, the recurrence of pancreatitis upon rechallenge with dapagliflozin and the resolution of symptoms after dapagliflozin cessation make it a likely cause of pancreatitis.

The underlying mechanism of dapagliflozin-induced pancreatitis is unclear. It is likely to be an idiosyncratic reaction similar to other cases of drug-induced pancreatitis, occurring as a result of immunological or cytotoxic effects of the drug or its metabolites on the body [22, 23].

In conclusion, this report highlights the possible association of SGLT-2 inhibitors and pancreatitis. Patients should be informed about the symptoms of acute pancreatitis and advised to discontinue SGLT-2 inhibitors in case such symptoms occur.

SGLT-2 inhibitors, although approved for the management of T2DM, should be prescribed with caution particularly in patients with increased risk for pancreatitis. Regulatory entities such as the FDA and Health Canada have identified a potential safety issue and possible link between SGLT-2 inhibitors and acute pancreatitis [24, 25]. Further studies are required to investigate the exact etiology of the reported adverse reaction to the medication.

**Figure 1:**
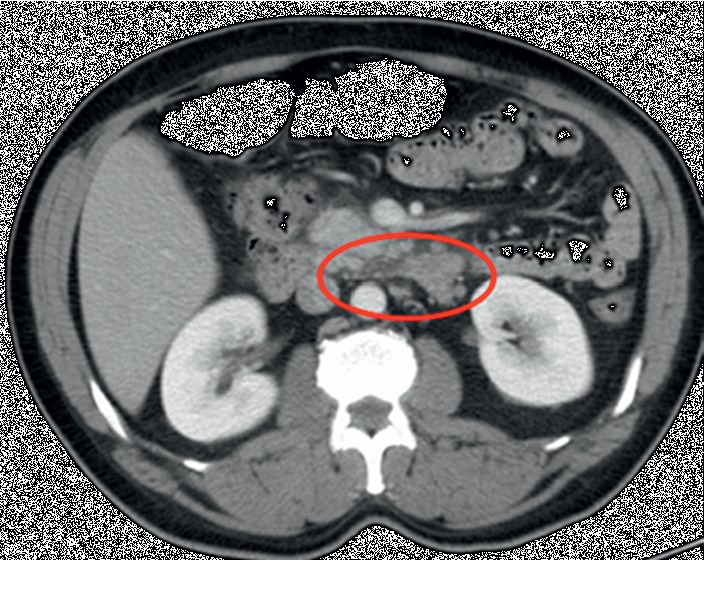
CT scan after initial presentation of acute pancreatitis.

**Figure 2:**
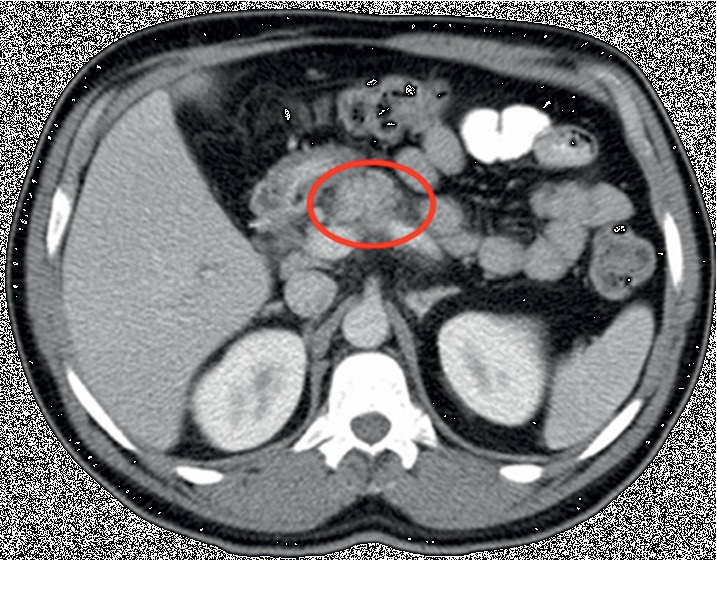
CT scan after subsequent episode of acute pancreatitis.

Figures 1 and 2 depict the patient's abnormal CT scan revealing findings of acute pancreatitis.

## Figures and Tables

**Table 1:** Published case reports for SGLT-2 inhibitor-induced pancreatitis in the literature.

Author	SGLT-2 inhibitor	Age/sex	Ethnicity	Other reported medications	Time from medication administration until pancreatitis	Severity (description) and outcome
Patel et al. [14]	Canagliflozin	71/F	Not specified	Metformin, sitagliptin	84 days	Necrotizing pancreatitis, resolved
Chowdhary et al. [15]	Canagliflozin	33/F	African-American	Metformin, levothyroxine	14 days	Severe acute pancreatitis, resolved
Srivali et al. [16]	Canagliflozin	50/M	White	Metformin, glyburide	10 days	Acute pancreatitis precipitating DKA, resolved
Verma [17]	Canagliflozin	46/F	White	Metformin, insulin, lisinopril, lovastatin, citalopram, estradiol, oxybutynin	21 days	Moderately severe acute pancreatitis, resolved
Gutch et al. [18]	Dapagliflozin	48/M	Indian	Metformin	7 days	Acute pancreatitis precipitating DKA, resolved
McIntire and Bayne [19]	Empagliflozin	70/M	African-American	Insulin, HCTZ, lisinopril, atenolol, ibuprofen, ranitidine, simvastatin, simethicone	104 days	Mild pancreatitis, resolved
Lightbourne et al. [20]	Empagliflozin	52/F	Not specified	Metformin, insulin, furosemide	30 days	Acute pancreatitis, resolved
